# Myotonia Congenita Mutation Enhances the Degradation of Human CLC-1 Chloride Channels

**DOI:** 10.1371/journal.pone.0055930

**Published:** 2013-02-12

**Authors:** Ting-Ting Lee, Xiao-Dong Zhang, Chao-Chin Chuang, Jing-Jer Chen, Yi-An Chen, Shu-Ching Chen, Tsung-Yu Chen, Chih-Yung Tang

**Affiliations:** 1 Department of Physiology, College of Medicine, National Taiwan University, Taipei, Taiwan; 2 Neuroscience Center, University of California Davis, Davis, California, United States of America; 3 Department of Medical Research, National Taiwan University Hospital, Taipei, Taiwan; 4 Graduate Institute of Brain and Mind Sciences, College of Medicine, National Taiwan University, Taipei, Taiwan; University of Houston, United States of America

## Abstract

Myotonia congenita is a hereditary muscle disorder caused by mutations in the human voltage-gated chloride (Cl^−^) channel CLC-1. Myotonia congenita can be inherited in an autosomal recessive (Becker type) or dominant (Thomsen type) fashion. One hypothesis for myotonia congenita is that the inheritance pattern of the disease is determined by the functional consequence of the mutation on the gating of CLC-1 channels. Several disease-related mutations, however, have been shown to yield functional CLC-1 channels with no detectable gating defects. In this study, we have functionally and biochemically characterized a myotonia mutant: A531V. Despite a gating property similar to that of wild-type (WT) channels, the mutant CLC-1 channel displayed a diminished whole-cell current density and a reduction in the total protein expression level. Our biochemical analyses further demonstrated that the reduced expression of A531V can be largely attributed to an enhanced proteasomal degradation as well as a defect in protein trafficking to surface membranes. Moreover, the A531V mutant protein also appeared to be associated with excessive endosomal-lysosomal degradation. Neither the reduced protein expression nor the diminished current density was rescued by incubating A531V-expressing cells at 27°C. These results demonstrate that the molecular pathophysiology of A531V does not involve anomalous channel gating, but rather a disruption of the balance between the synthesis and degradation of the CLC-1 channel protein.

## Introduction

Myotonia congenita, a hereditary muscle disorder caused by mutations in the human *CLCN1* gene on chromosome 7 [1], is characterized by muscle stiffness after voluntary contraction. The gene *CLCN1* encodes a voltage-gated chloride (Cl^−^) channel, CLC-1, which is nearly exclusively expressed in skeletal muscles [2]. It has been estimated that CLC-1 channels may contribute up to 70%–80% of the resting membrane conductance of the skeletal muscle [3], [4], [5] and therefore play a pivotal role in controlling the excitability of sarcolemma membranes. The CLC-1 channel contains two identical pores (also called protopores), suggested first by the “double-barreled” opening of functional channels [6], [7], [8], and later by the recent structural findings that two identical Cl^–^transport pathways are present in one CLC protein [9], [10], [11]. The opening and closing of the two pores in CLC-1 channels are controlled by two distinct gating mechanisms [12]: the “common-gate” that controls the opening and closing of two protopores simultaneously and the “fast-gate” that controls each individual protopore and operates independently from the partner fast-gate.

So far, more than 100 different mutations in the *CLCN1* gene have been identified in patients with myotonia congenita [13], [14], [15]. These various myotonia mutations can be inherited in an autosomal recessive (Becker type) or dominant (Thomsen type) fashion [16]. The molecular basis for the inheritance pattern of myotonia congenita has been explained by the consequence of the mutation on the gating of CLC-1 channels: those mutations that affect the common-gate lead to an autosomal dominant inheritance, whereas those affecting individual fast-gates only result in a recessive pattern [6], [17]. Indeed, a dominant negative effect on the common gating of CLC-1 appeared to explain the dominant inheritance of mutations that occurred at the dimer interface [18], [19]. Several recessive *CLCN1* mutations, however, have been shown to yield functional CLC-1 channels with gating properties either only slightly different or virtually indistinguishable from those of wild-type (WT) channels [14]. Similarly, some dominant *CLCN1* mutations display no detectable gating defects upon forming hetero-dimers with their WT counterparts [20]. These examples suggest that the effects of myotonia-related mutations entail mechanisms other than the disruption of CLC-1′s gating functions. Indeed, studies of the epitope-tagged CLC-1 proteins expressed in *Xenopus* oocytes have revealed that a reduced surface expression of CLC-1 channels may be the underlying pathology of some myotonia mutations [21]. A reduced protein expression in cell’s surface membranes has also been documented in other ion channels. For example, a majority of cystic fibrosis patients suffer from a defect in the maturation and membrane trafficking of the cystic fibrosis transmembrane regulator (CFTR) caused by a phenylalanine deletion mutation, ΔF508 [22], [23].

In this report, we examine a myotonia congenita-associated CLC-1 mutation, A531V [24]. It has been suggested that A531V displays impaired protein stability [25]; but the mutant channel has not been functionally characterized, nor has the expression level of this mutant in the cell membrane been examined. Our electrophysiological analyses indicate that the A531V mutant channel has gating properties similar to those of the WT channel but yields dramatically diminished whole-cell currents. Biochemical studies further reveal that the reduction in whole-cell currents of the A531V mutant results from significantly enhanced protein degradation. Our data imply that most of the mutant protein may fail to pass the quality control system for the biosynthesis of CLC-1 proteins.

## Results

### A531V Produces Functional CLC-1 Channels with Significantly Reduced Current Density

**Figure 1:**
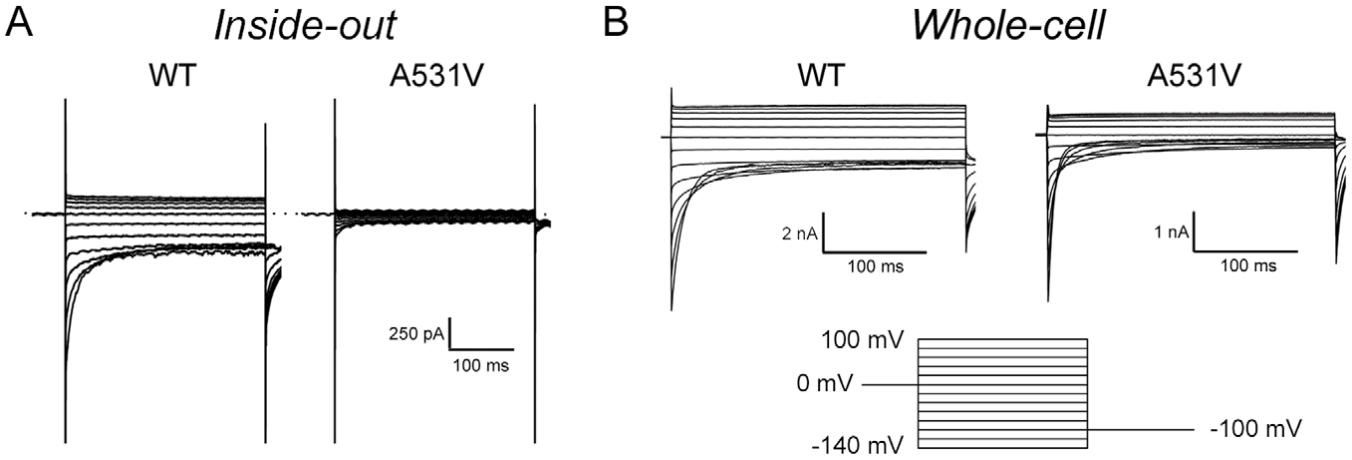
Expression of the WT CLC-1 and the A531V mutant. *(* ***A*** *)* Excised inside-out and *(* ***B*** *)* whole-cell patch-clamp recordings of the WT CLC-1 channel and the A531V mutant in tsA201 cells. The voltage protocol is shown in the *lower panel*: the membrane potential was first stepped from a holding potential of 0 mV to various test-voltages from +100 mV to −140 mV in −20 mV steps for 300 ms, followed by a tail-voltage step to −100 mV for 300 ms.

**Figure 2:**
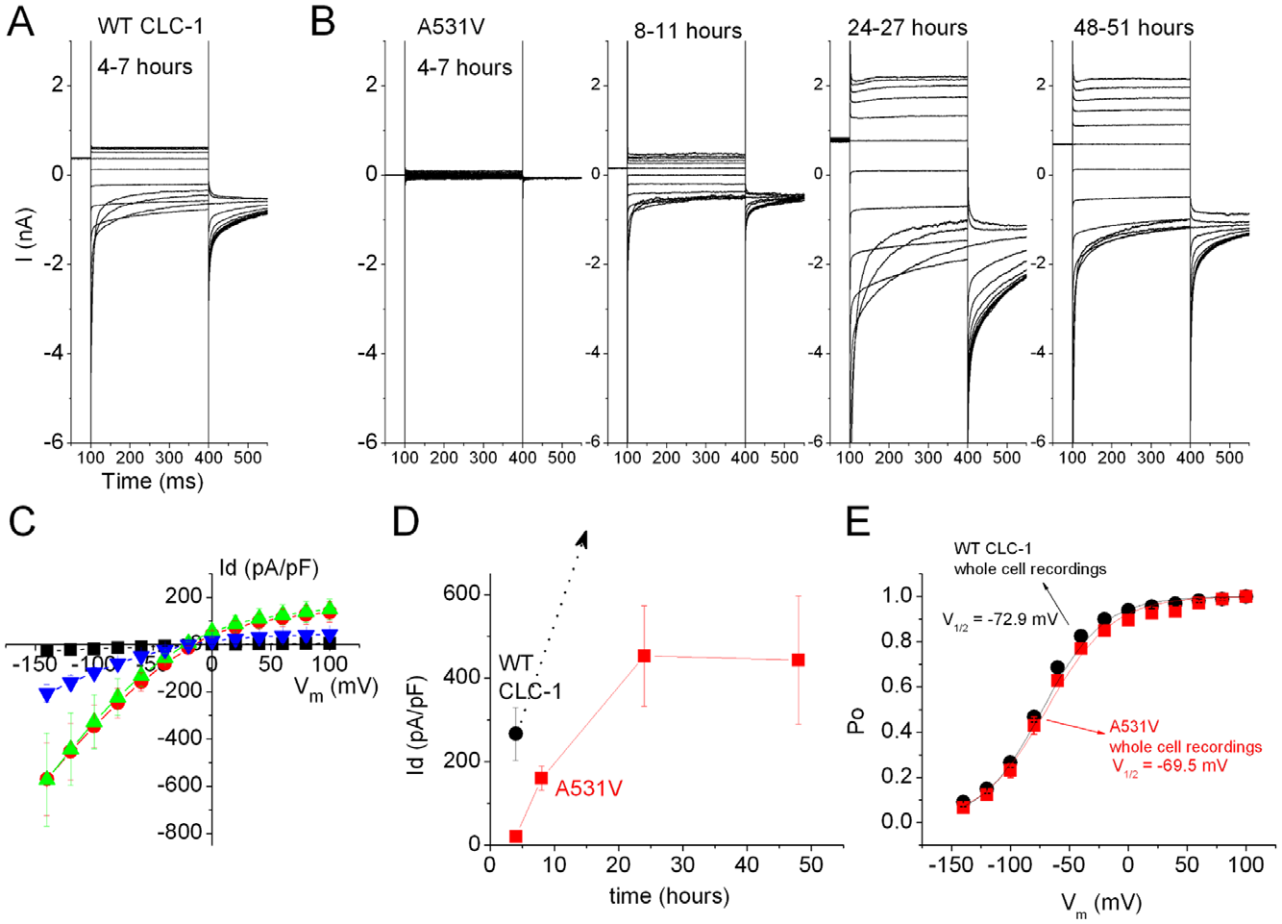
Comparison of the current density as a function of time for the WT CLC-1 and the A531V mutant. All data were obtained from whole-cell patch-clamp recordings in tsA201 cells. *(* ***A*** *)* Recording of WT CLC-1 4–7 hrs after transfection. *(* ***B*** *)* Recordings of the A531V mutant at the indicated time periods after transfection. *(* ***C*** *)* Averaged instantaneous current-voltage (I-V) curves of the A531V mutant during the four time periods indicated in B. Current amplitude is shown in the form of current density (Id; whole cell current/cell capacitance). Colors of the symbol represent: Black, 4–7 hrs (n = 14); Blue, 8–11 hrs (n = 7); Green, 24–27 hrs (n = 5); Red, 48–51 hrs (n = 4). *(* ***D*** *)* Current density of WT and A531V as a function of time after transfection. The instantaneous current at Vm = −120 mV was used for the calculation. The dotted arrow for WT (black color) represents the fact that the cells were un-clampable at 24 hrs after transfection. *(* ***E*** *)* Steady-state *P*_o_–V curves of the WT CLC-1 and the A531V mutant.

We began our study by performing excised inside-out patch-clamp recordings to evaluate the functional properties as well as the expression level of the channels in tsA201 cells. As shown in Figure 1A, membrane patches from cells expressing the A531V mutant exhibited a very small CLC-1-like current in comparison with those from the cells expressing the WT CLC-1 channel. To further evaluate the channel expression level, we also used whole-cell recordings to compare the WT channel versus the A531V mutant. Figure 1B illustrates representative whole-cell recordings of WT and A531V: the mutant channel indeed displayed significant CLC-1 currents. In order to more closely compare the functional expression level of WT and A531V channels, we decided to perform whole-cell recordings at various post-transfection time points. Figure 2A demonstrates representative whole-cell CLC-1 current traces recorded from WT channels 4–7 hours post-transfection. In contrast, no significant whole-cell current was observed for A531V until 8–11 hours post-transfection (Fig. 2B). The current amplitude of A531V reached a steady-state level at about 24 hours post-transfection (Fig. 2C–D), and the estimated whole-cell current density of A531V was significantly smaller than that of WT (Fig. 2D). Nonetheless, the steady-state I-V curve and the *P_o_*-V curve of A531V were similar to those of the WT CLC-1 (Fig. 2D–E). These results for the first time demonstrate that the A531V mutant appears to have similar functional properties as the WT channel.

**Figure 3:**
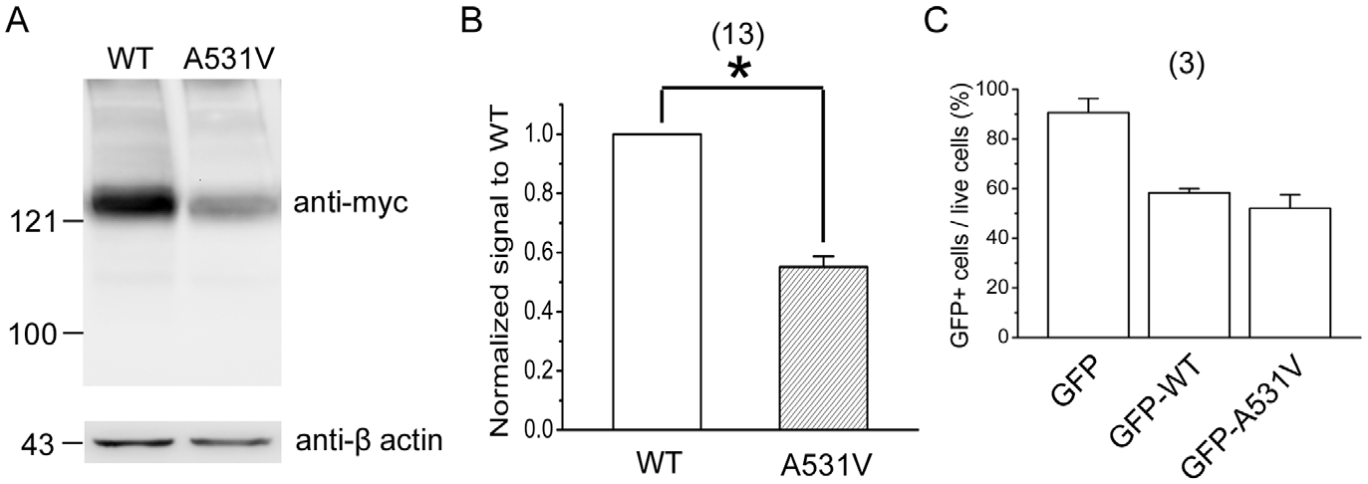
Total protein expression of WT and A531V CLC-1 channels. Biochemical analyses of CLC-1 channels expressed in HEK293T cells. *(* ***A*** *)* Immunoblotting analyses of myc-tagged WT CLC-1 and the A531V mutant. The position of molecular weight markers (in kilodaltons, kDa) are at the left of the blots. Expressions of β-actin are displayed as controls for the loaded protein amounts. *(* ***B*** *)* Quantification of CLC-1 protein expression level. Protein density was standardized as the ratio of the myc-CLC-1 signal to the cognate β-actin signal. Values from the A531 mutant were then normalized to those for WT. Densitometric scans of immunoblots were obtained from 13 independent experiments. The mean normalized value of A531V is 0.57±0.02. Asterisks denote significant difference from WT (*, *t*-test: p<0.05). (***C***) Quantification of the percentage of transfected HEK293T cells emitting GFP fluorescence (GFP+/live cells). Flow cytometry was employed to determine the ratio for each of the three listed cDNA constructs. Data were pooled from 3 independent experiments.

The difference in current densities can be explained at least in part by the different levels of total channel protein expression. As depicted in Figure 3A showing the HEK293T cells over-expressing myc-tagged CLC-1 proteins, the immunoreactivity of A531V was significantly less than that of WT. Quantification of the total protein amount revealed that the protein expression level of the A531V mutant was only ∼60% of that of the WT channel (Fig. 3B). Furthermore, flow cytometric analyses of HEK293T cells transfected with the GFP-tagged WT or A531V channels revealed no discernible difference in the percentage of cells emitting GFP fluorescence (Fig. 3C), indicating that the remarkable decrease in the A531V expression level was unlikely a result of diminished DNA transfection efficiency. Together these data strongly suggest that the low current amplitude of A531V is more likely due to poor channel expression rather than abnormal functional properties.

### A531V is Subject to Enhanced Protein Degradation Mediated by Proteasome

**Figure 4:**
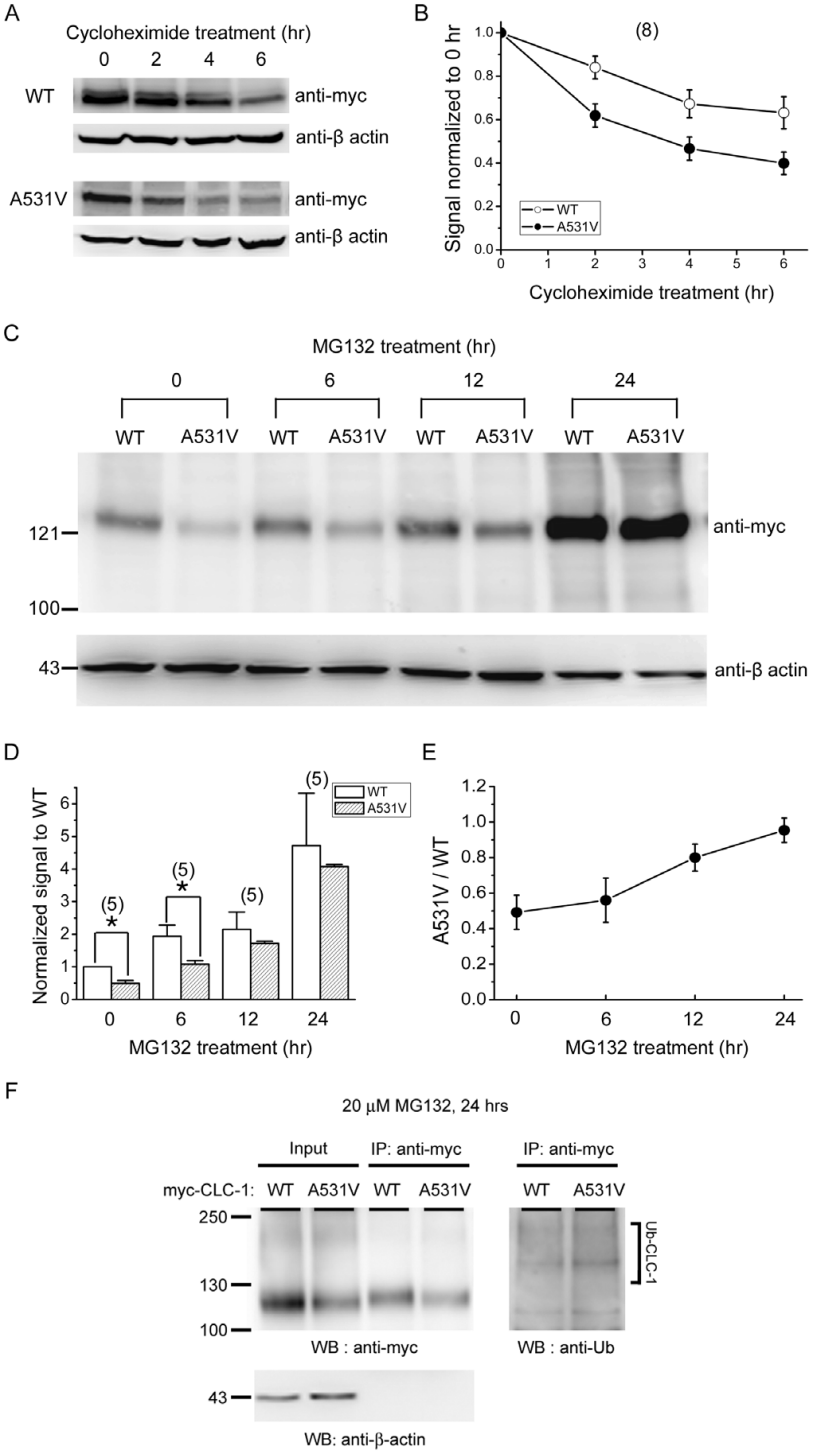
Rescue of A531V protein expression with a proteasomal degradation blocker. Characterization of CLC-1 protein turn-over in HEK293T cells. *(* ***A*** *)* Kinetics of protein degradation for WT CLC-1 and A531V in the presence of cycloheximide (100 µg/ml). *(* ***B*** *)* Quantification of CLC-1 protein expression levels in response to different cycloheximide treatment durations. Protein densities were standardized as the ratio of the myc-CLC-1 signal to the cognate β-actin signals, followed by normalization to those of the control at 0 hr. Data were averaged from 8 independent experiments. *(* ***C*** *)* The effect of treatment with 20 µM MG132. *(* ***D*** *)* Quantification of CLC-1 protein expression levels in response to different MG132 treatment durations. The scanned intensities of protein densities were normalized to those of WT with no drug treatment. *(* ***E*** *)* The relative expression ratio of A531V with respect to WT (as calculated from D) was plotted against the duration of the MG132 treatment. *(* ***F*** *)* Ubiquitination of CLC-1 proteins. Transfected cells were incubated at 37°C for 24 hrs in the presence of MG132. Cell lysates were immunoprecipitated (IP) with the anti-myc antibody, followed by immunoblotting (WB) with the anti-myc or anti-ubiquitin (Ub) antibody. Corresponding expression levels of CLC-1 constructs in the lysates are shown in the *Input* lane, which represents 5% of the total protein used for immunoprecipitation. Ub-CLC-1: ubiquitinated CLC-1.

The net expression level of any channel protein depends in theory on a balance between protein synthesis and protein degradation. A decrease in protein synthesis, as well as an increase in protein degradation, could contribute to the low expression of A531V. We first explored the possibility that the A531V mutation may accelerate the degradation of channel proteins because a previous pulse-chase study in L6 myotube cells suggested that the A531V mutant may suffer from decreased protein stability [25]. To more rigorously address the protein stability problem, we compared the protein half-life of the WT and the mutant channel. As shown in Figures 4A–B, at 2 hours after the treatment of 100 µg/ml cycloheximide, a protein synthesis inhibitor, A531V protein was decreased by ∼40%, in comparison to ∼15% diminution observed for its WT counterpart. Linear-regression analyses of the time course of protein degradation with up to 6 hours of cylcoheximide treatment revealed that the protein half-life for WT and A531V was about 7.6 and 3.7 hours, respectively, a notable reduction for the mutant channel.

One important proteolysis mechanism during the early biosynthesis process of proteins is the clearance of misfolded proteins by proteasomes. Peptide aldehydes such as MG132 are commonly used to examine the involvement of this mechanism in mammalian cells [26], [27]. It has been shown that treating cells by up to 50 µM of MG132 for 10–24 hours exerts an effective proteasome inhibition without significantly affecting cell viability [27], [28], [29], [30]. We thus employed 20 µM MG132 to assess the role of the proteasomal degradation in the low expression of A531V. As depicted in Figure 4C, 20 µM MG132 displayed a significant time-dependent enhancement of the total protein level for both WT and A531V. More importantly, the disparity in total protein expression between WT and A531V became less prominent as the duration of the MG132 treatment increased, and no significant difference was observed after 12- to 24-hours of treatment (Fig. 4D–E). Proteasomal degradation is known to be preceded by protein ubiquitination [31], [32]; in agreement with this notion, we observed in HEK293T cells that both WT and A531V proteins were significantly ubiquitinated (Fig. 4F). This biosynthetic anomaly was unlikely to be caused by HEK cell-specific artifacts, since a similar reduction of protein expression and recovery by MG132 were observed in COS-7 cells transfected with the A531V construct (Fig. S1). Taken together, these results suggest that the defective expression of A531V may result from enhanced proteasomal degradation.

### MG132-rescued A531V Protein Displays Reduced Membrane Surface Expression

**Figure 5:**
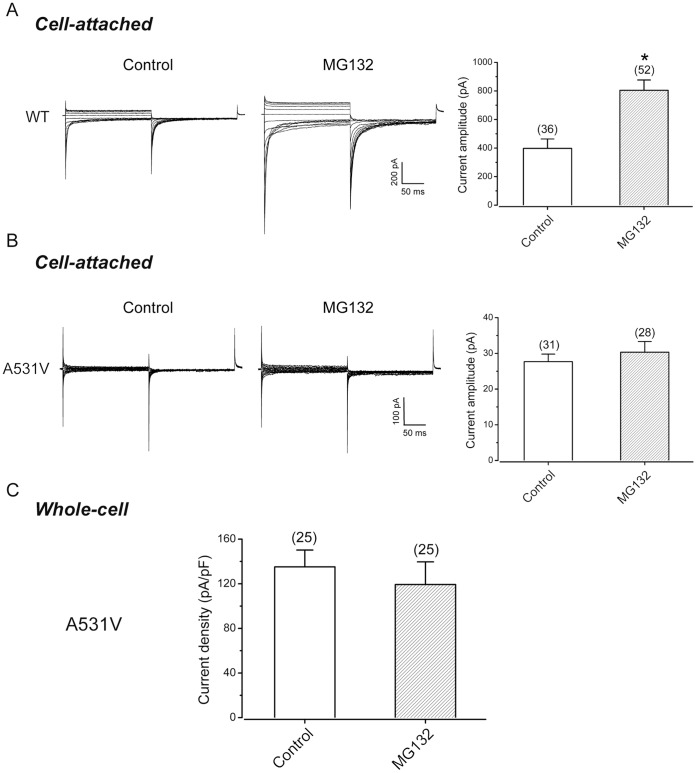
MG132 treatment fails to rescue the functional expression of A531V. *(* ***A & B*** *)* Effects of the MG132 treatment (20-µM, 24 hrs) on the functional expression of WT and A531V in HEK293T cells. *Left and middle panels* are cell-attached patch recordings from cells with and without the MG132 treatment, respectively. The averaged current amplitudes were compared in the *right panels* with the number of patches shown on top of each column. The asterisk denotes a significant difference from the control (no MG132 treatment) condition (*, *t*-test: p<0.05). *(* ***C*** *)* Effects of the MG132 treatment on the current density of A531V channels. Data were derived from whole-cell recordings. The instantaneous currents at Vm = -140 mV were used for the calculation.

If MG132 treatment can rescue the defective total protein expression, will the same treatment also restore the reduced current density of A531V as assayed by electrophysiological recordings? Figures 5A–B exemplify the effects of MG132 treatment on the functional expression of the WT channel and the A531V mutant, respectively. The current amplitude of WT CLC-1 channels averaged from 30–50 cell-attached patches doubled after 24 hours of the MG132 treatment (Fig. 5A), consistent with the aforementioned upsurge of CLC-1 protein expression after proteasome inhibition. Surprisingly, despite an increase of the total protein level of A531V by the MG132 treatment, no significant increase in Cl^−^ current was observed in patches recorded from the A531V-transfected cells (Fig. 5B). Whole-cell recordings of the mutant channels in HEK293T cells further confirmed that the treatment of MG132 failed to boost the current density of the A531V-transfected cells (Fig. 5C).

**Figure 6:**
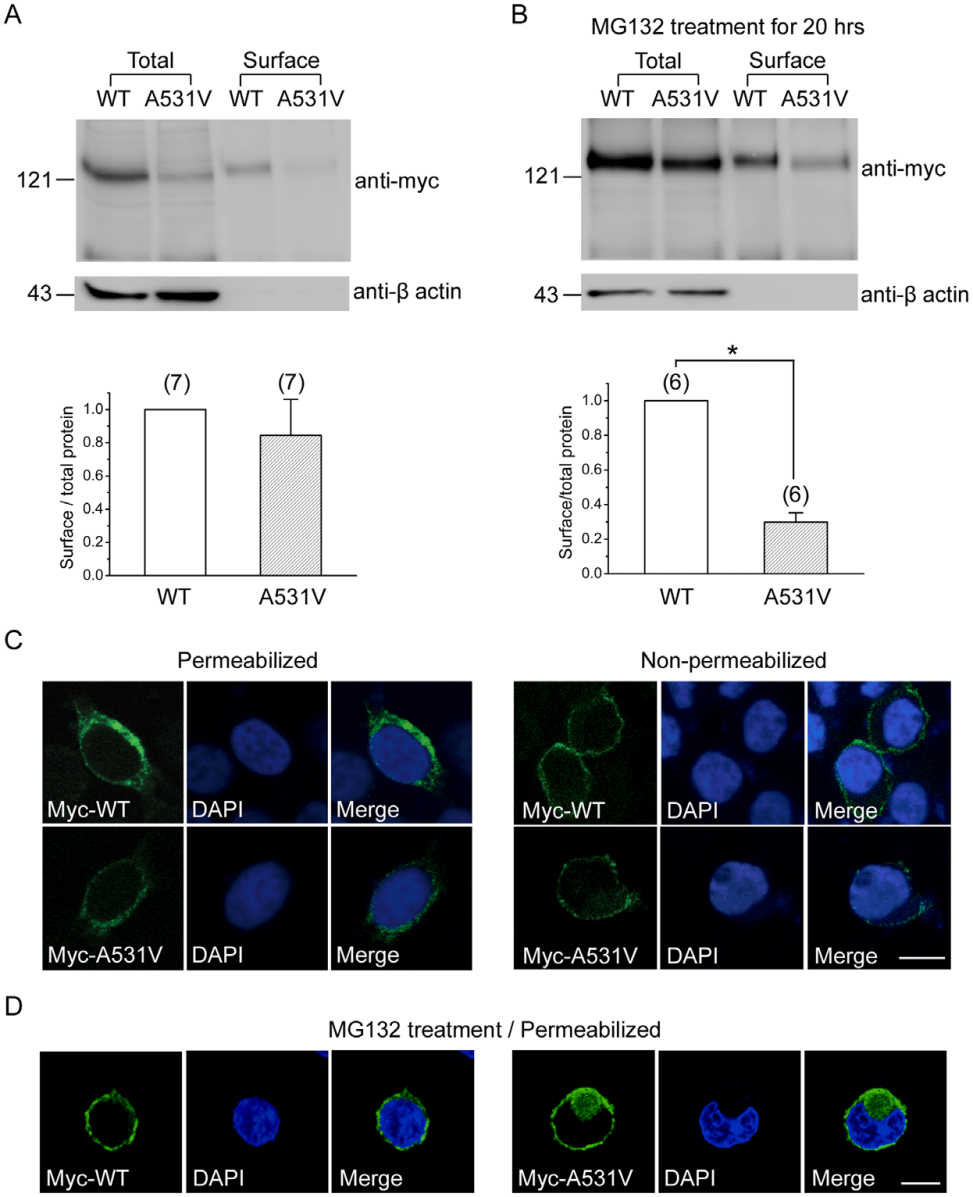
Surface expression efficiency of WT and A531V channels. Surface biotinylation experiments on HEK293T cells expressing myc-tagged CLC-1 channels in the absence *(* ***A*** *)* or presence *(* ***B*** *)* of 24-hr treatment with 20 µM MG132. (*Total*) Cell lysates were directly employed for immunoblotting analyses. (*Surface*) Cell lysates were from biotinylated intact cells, after pulling down with streptavidin beads. To quantify the surface expression efficiency (*lower panels*), the total protein density was standardized as the ratio of input signal to β-actin signal. The efficiency of surface presentation was expressed as surface protein density divided by the corresponding standardized total protein density. The mean surface expression ratio of the A531V mutant was normalized to that of WT. Densitometric scans of immunoblots were obtained from six to seven independent experiments. *(* ***C,D*** *)* Confocal microscopic images of HEK293T cells expressing myc-tagged CLC-1 channels in the absence *(* ***C*** *)* or presence *(* ***D*** *)* of the MG132 treatment. Fixed cells were stained with the anti-myc antibody (*left panels*) as well as the nuclear counterstain DAPI (*middle panels*) under the permeabilized or non-permeabilized configuration. Scale bar = 10 µm.

One explanation for this seemingly paradoxical effect of MG132 on A531V is that the mutant proteins rescued by the proteasome inhibitor may be defective in the membrane trafficking process. We therefore utilized the biotinylation technique to quantitatively compare the surface expression efficiency of WT and A531V. Figure 6A shows that in the absence of MG132, the surface expression ratio of A531V was comparable to that of WT. In response to the MG132 treatment, however, the surface expression efficiency of A531V severely deteriorated, only about 30% of that for WT (Fig. 6B). In addition, we studied the effect of the MG132 treatment on the subcellular localization pattern of the mutant channel. In the absence of the proteasome inhibitor, the majority of myc-tagged A531V displayed a ring-shaped fluorescence signal along the cell perimeter, as exemplified by the confocal microscopic image of permeabilized HEK293T cells in Figure 6C. Immunofluorescence analyses of intact, non-permeabilized HEK293T cells further confirmed that myc-tagged A531V channels could be detected by the anti-myc antibody applied extracellularly (Fig. 6C). In response to the MG132 treatment, however, we observed a significant cytoplasm-localization pattern for the mutant channel (Fig. 6D). Altogether these data imply that after the MG132 treatment, the majority of the A531V protein spared from proteasomal degradation is still rejected from the membrane trafficking pathway, thereby failing to form functional channels in the plasma membrane.

### A531V is Associated with Excessive Endosomal-lysosomal Proteolysis

**Figure 7:**
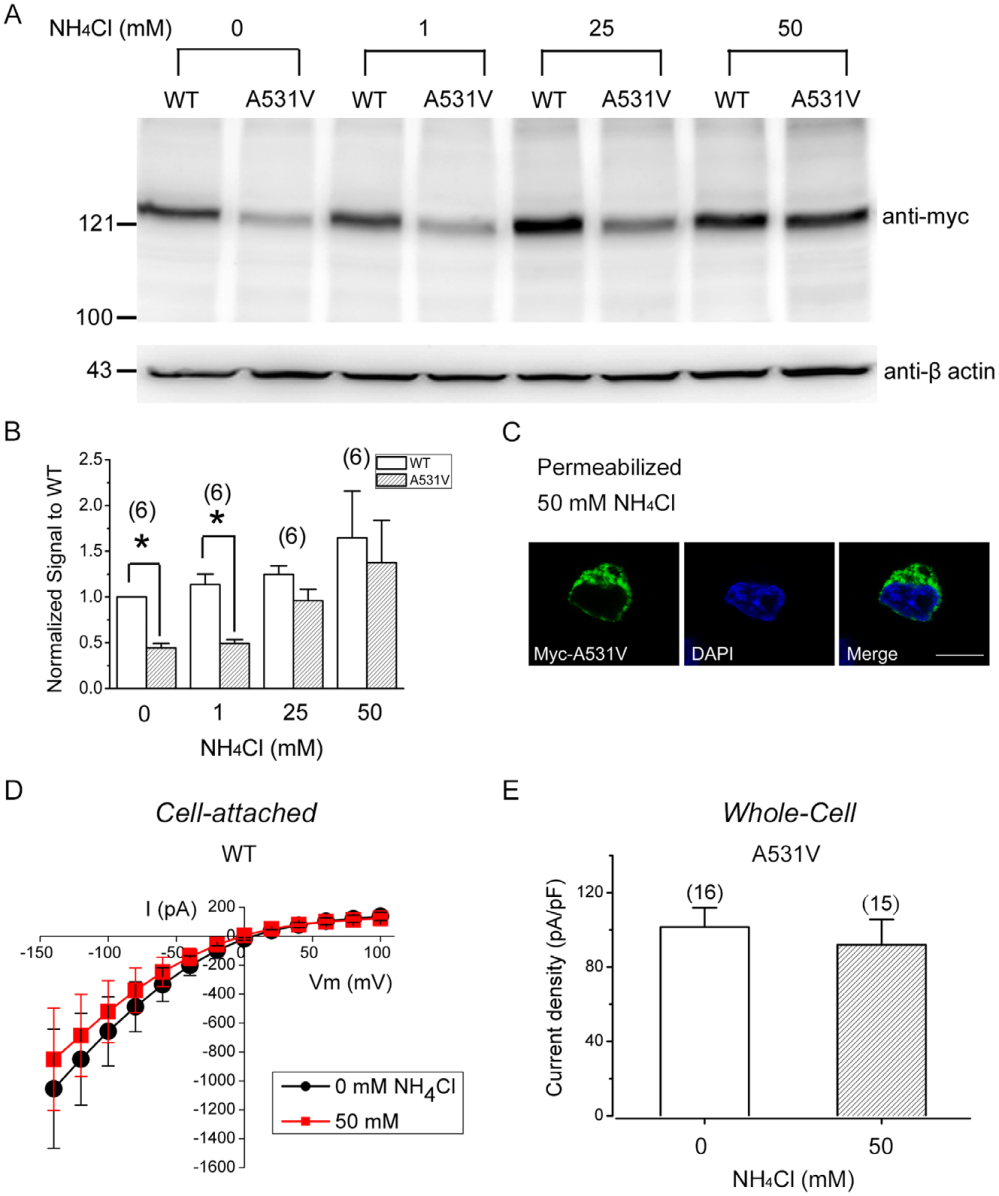
Effects of blocking the endosomal-lysosomal degradation pathway. *(* ***A*** *)* Immunoblotting analyses of cell lysates from CLC-1-expressing HEK293T cells subject to treatment with increasing concentrations of NH_4_Cl for 24 hrs. *(* ***B*** *)* Quantification of CLC-1 protein expression levels in response to 24-hr treatment with different NH_4_Cl concentrations. Protein densities were normalized with respect to those for WT with no drug treatment by following the same procedure as described in Figure 4B. *(* ***C*** *)* Immunofluorescence images of HEK293T cells expressing myc-tagged A531V channels in the presence of 50 mM NH_4_Cl for 24 hrs. Cells were fixed under the permeabilized configuration. Scale bar = 10 µm. *(* ***D*** *)* Averaged instantaneous I-V curves of WT CLC-1 recorded under the cell-attached configuration. HEK293T cells were incubated in the absence (*black circles*; n = 15) or presence (*red squares*; n = 17) of 50 mM NH_4_Cl for 24 hrs. *(* ***E*** *)* Whole-cell current density (at −140 mV) of the A531V mutant recorded from HEK293T cells with or without the NH_4_Cl treatment.

Recent studies indicate that in addition to proteasomal degradation, several misfolded CFTR mutants are also subject to an endosomal-lysosomal degradation [33], [34]. We therefore explored the potential contribution of the endosomal-lysosomal pathway by utilizing NH_4_Cl, a weak base that elevates the pH of the endosomal-lysosomal compartment, thereby inhibiting endosomal-lysosomal protein degradation [30], [35]. Application of up to 50 mM NH_4_Cl for 24 hours, which failed to induce significant cell damage [30], [35], only slightly increased the protein level of WT channels (Fig. 7A–B). By contrast, the same NH_4_Cl treatment led to a notable enhancement in the total protein level of the A531V mutant (Fig. 7A–B). Importantly, in the presence of 25 or 50 mM NH_4_Cl, the difference between the protein expressions of A531V and WT became statistically insignificant (Fig. 7B), suggesting that a significant fraction of the A531V protein may be susceptible to excessive endosomal-lysosomal proteolysis. Accordingly, immunofluorescence analyses of permeabilized HEK293T cells revealed a significant cytoplasmic punctuate staining pattern for the mutant channel in response to the NH_4_Cl treatment (Fig. 7C). We also examined the effect of 50 mM NH_4_Cl on the functional expression of CLC-1 channels. Consistent with the foregoing biochemical observation, the NH_4_Cl treatment did not notably affect the current amplitude of WT CLC-1 channels under the cell-attached configuration (Fig. 7D). Furthermore, despite an enhancement of the total protein level of A531V in the presence of 50 mM NH_4_Cl, we observed no significant difference in the whole-cell current density (Fig. 7E). Therefore, similar to the effect of the MG132 treatment, inhibition of the endosomal-lysosomal pathway does not facilitate the functional expression of the A531V mutant.

### Low Temperature Incubation Fails to Rescue the Biosynthetic Anomaly of A531V

**Figure 8:**
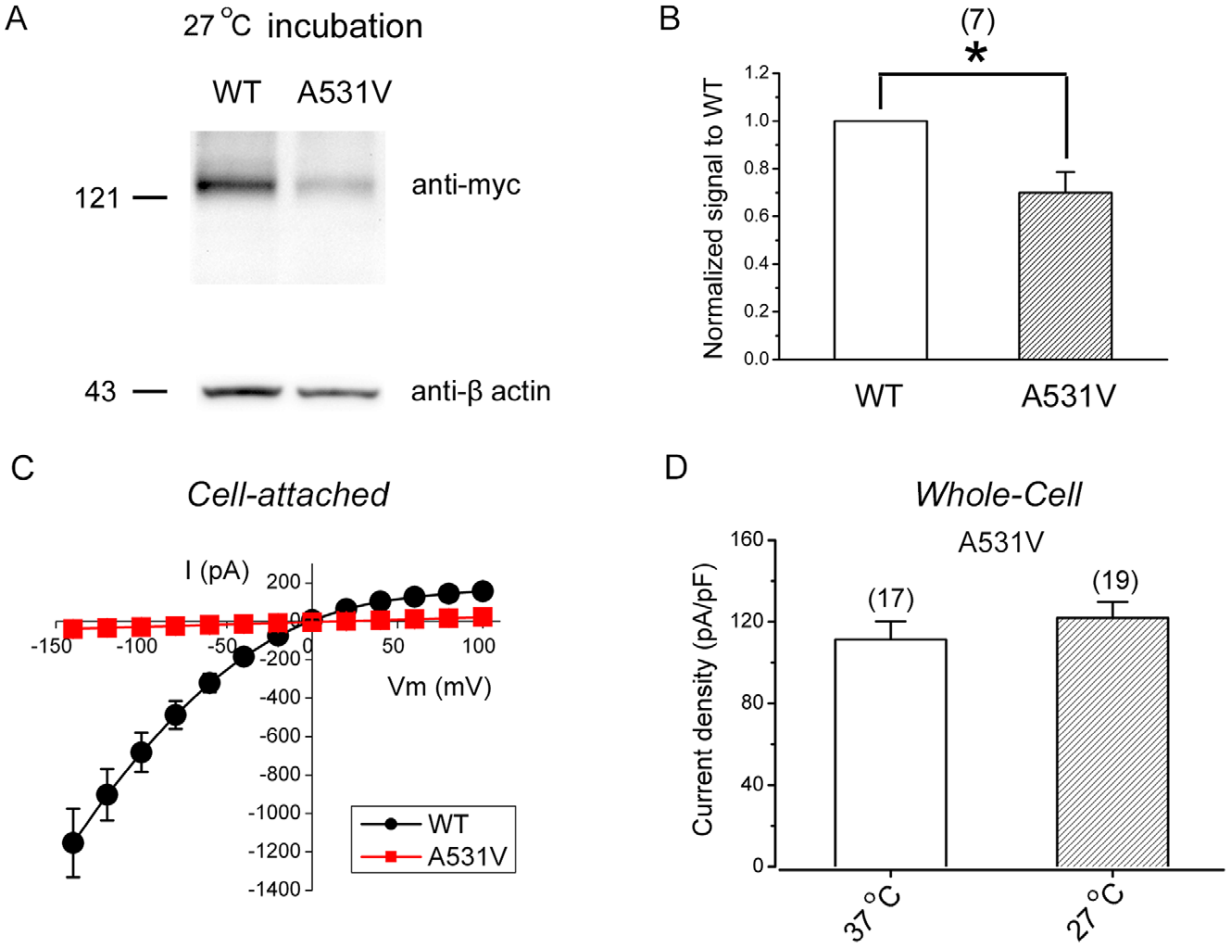
The biosynthetic anomaly of A531V is temperature-insensitive. *(* ***A*** *)* Immunoblotting analyses of myc-tagged WT and A531V CLC-1 proteins from HEK293T cells incubated at 27°C for 48 hrs. *(* ***B*** *)* Quantification of total protein expression level by following the same procedure as described in Figure 4B. Data were obtained from 7 independent experiments. The mean normalized value of A531V is 0.70±0.09. Asterisks denote significant difference from WT (*, *t*-test: p<0.05). *(* ***C*** *)* Averaged instantaneous I-V curves of the WT (*black circles*; n = 36) and the A531V mutant (*red squares*; n = 26) recorded under the cell-attached configuration. CLC-1 channel-expressing HEK293T cells were incubated at 27°C for 48 hrs. *(* ***D*** *)* Whole-cell current density (at −140 mV) of the A531V mutant recorded from HEK293T cells incubated at 37°C or 27°C.

The trafficking defects of numerous disease-related mutant channels can be partially corrected following incubation at reduced temperature [22], [36], [37]. To address the potential temperature sensitivity of the A531V mutant, we incubated transfected HEK293T cells at 27°C for 48 hours prior to biochemical or functional analyses. Figures 8A–B demonstrate that reduced temperature had no discernible effect on the protein expression level of the mutant. In support of this biochemical assay, no significant Cl^−^ current was observed in patches recorded from the A531V-transfected cells incubated at 27°C (Figure 8C). Similarly, the whole-cell current density of A531V was not significantly enhanced following reduced temperature incubation (Figure 8D). Together, these data suggest that the biosynthetic anomaly of the A531V mutant is temperature-insensitive.

## Discussion

A531V is a myotonia congenita-associated mutation located at the helix O of the human CLC-1 channel. The A531V mutation is found in significant prevalence in northern Finland as well as northern Scandinavia [24], [38]. Although A531V was previously reported to display impaired protein stability in L6 myotubes [25], it was unclear whether the observed instability represented proteasomal and/or endosomal-lysosomal degradation of the mutant protein. Moreover, given that virtually no heterologously expressed WT CLC-1 protein was properly exported to the membrane in L6 myotubes, the foregoing study was unable to functionally characterize the mutant channel. The same research group also investigated the subcellular localization of A531V heterologously expressed in isolated rat myofibers, and observed a significant endoplasmic reticulum (ER)-retention for the mutant CLC-1 channel [25]. Nevertheless, the authors did not quantitatively analyze either the total protein production or the functional expression of the A531V mutant in myofibers.

In the present report, we have functionally and biochemically characterized the A531V mutation. This CLC-1 mutant displays a dramatically diminished whole-cell current density, a striking reduction in the total protein expression level, and a significantly shorter protein half-life. The reduced protein expression of A531V is largely rescued by the proteasome inhibitor MG132, consistent with enhanced proteasomal protein degradation of the A531V mutant. Importantly, even after the inhibition of proteasomal degradation by the MG132 treatment, the majority of the A531V protein is prohibited from reaching the cell membrane, implying that most of the mutant protein fail to pass the ER quality control system and consequently display defective membrane trafficking. In addition, we have presented evidence suggesting that the mutant is subject to significant endosomal-lysosomal degradation as well. We thus propose that A531V is endowed with a folding anomaly that makes the mutant channel undesirable for the protein quality control system in ER (and perhaps plasma membrane), thereby leading to a bias of the biosynthetic balance tilted toward the degradation pathway.

Defective membrane trafficking is frequently found in disease-associated mutant ion channels. However, in addition to the membrane trafficking defect, the ΔF508 mutant of CFTR also showed altered gating kinetics [39], [40], [41]. The function of the A531V mutant of CLC-1 has never been studied before. Here we employed various patch-clamp recording methods to examine the function as well as the expression level of the A531V mutant. We have demonstrated that the *P_o_*-V curve and the I-V relationship of the A531V mutant are similar to those of the WT channel. Therefore, the major defects of this myotonia mutation appear to occur mainly in the biosynthesis of the channel protein.

It remains unclear how the A531V mutation renders most of the mutant protein unacceptable for the ER quality control system. One possibility is that the mutation A531V may disrupt the structure of the CLC-1 protein (or an ER-export signal) in a subtle manner without affecting its biophysical properties. Alternatively, the mutation may result in the exposure of an ER-retention signal as reported in many other ion channels. In either case, the abnormal structure of the mutant protein may serve as a trigger for the ER quality control system to redirect the biosynthesis process toward protein degradation [42], [43], [44]. We have searched ER-retention or ER-export signals found in potassium channels, glutamate receptors, CFTR, or other membrane proteins [45], [46], [47], [48], including sequences such as RXR, KKXX and VXXSL. Within residues 511–551 of CLC-1, we failed to identify any hint suggesting that an introduction of valine at position 531 would disrupt/generate any of these signals. It should be mentioned that all known ER-retention/export signals are located in the cytoplasmic domain, whereas A531 is presumably in the transmembrane helix region [9].

ER quality control mechanisms work in a stringent way to selectively remove misfolded proteins, a process known as ER-associated degradation (ERAD) [31], [32], thereby ensuring that the majority of proteins synthesized are structurally correct and functionally normal. It is believed that at least ∼30% of all newly-synthesized proteins from various cell types are degraded by proteasomes [49]. For instance, as much as 75% of the WT CFTR may fail to exit ER in heterologous expression systems [50]. Moreover, it has been estimated that 80% –90% of the protein degradation occurs by the proteasome pathway in most cultured mammalian cells [27]. We speculate that at least in the heterologous expression system newly synthesized CLC-1 proteins may be intrinsically inefficient in forming a correct structure that can pass the scrutiny of ER quality control mechanisms. It is therefore conceivable that a minor point mutation–such as A531V–may dramatically aggravate this intrinsic problem of CLC-1 proteins, thus rendering a substantial portion of A531V proteins unsuitable for entering the membrane trafficking pathway. Our demonstration of enhanced proteasomal degradation in the mutant strongly suggests that the majority of the newly synthesized A531V proteins are directed toward the ERAD pathway.

Under *in vitro* conditions, lowering of the incubation temperature to 26°C or 27°C partially corrected the trafficking defects of numerous disease-related mutant channels [22], [36], [37], giving rise to detectable ionic currents and mature proteins in electrophysiological and immunoblotting analyses, respectively. The mechanism of low temperature effects is not well understood and may involve improved protein folding, inhibition of proteasomal degradation, or increased surface trafficking. For a subset of mutant channels, however, their faulty protein maturation, trafficking defect, or proteasomal degradation are temperature-insensitive [37], [51]. In accordance with this precedence, the lack of effect of 27°C-incubation on the biosynthetic anomaly of A531V suggests that reduced temperature fails to correct the folding defect of the CLC-1 channel and thus cannot prevent the mutant protein from entering the ERAD pathway.

Both our functional and immunofluorescence data clearly demonstrate that a considerable portion of A531V protein manages to escape the ER quality control system and reach the plasma membrane. Misfolded membrane-bound proteins, however, are also susceptible to substantial degradation by the endosomal-lysosomal pathway, a mechanism known as the “peripheral quality control”. For example, proteolysis of CFTR mutants exported to the membrane has been shown to involve an ubiquitin-dependent targeting for the endosome-lysosome system [33], [34], [52]. That the NH_4_Cl treatment significantly increases the total protein level of the A531V mutant raises an intriguing possibility that a substantial portion of surface A531V protein may also fail the peripheral quality control and hence go through an enhanced ubiquitin-dependent endocytosis, eventually destined for endosomal-lysosomal degradation. In contrast, the slight increase in WT proteins in response to the NH_4_Cl treatment is consistent with the notion that the endosomal-lysosomal proteolysis may only contribute to the basal turn-over of WT CLC-1 channels in the plasma membrane. The A531V mutant may therefore serve as a useful tool in the future for deciphering the detailed mechanisms underlying the quality control of surface proteins.

An important issue that requires scrutiny is whether the foregoing working model on the molecular pathophysiology of A531V is only observed in the non-muscle heterologous expression system, or whether it can be applied to muscle cells. One piece of evidence supporting the latter came from the previous pulse-chase labeling experiment showing that when heterologously expressed in L6 myotubes, A531V displayed a defect in protein stability [25], consistent with our biochemical observations in the heterologous expression system. Furthermore, the same study observed a significant ER-retention for the mutant CLC-1 channel in isolated rat myofibers, implying that the A531V mutant is also unacceptable for the ER quality control system in myofibers. Therefore, we propose that enhanced protein degradation may indeed contribute to the pathogenesis of the A531V mutation in muscle cells. It will be interesting in the future to determine whether a similar scenario in biosynthetic anomaly may also apply to other myotonia mutations, especially for those CLC-1 mutants showing reduced functional and/or protein expression [20], [21].

Emerging evidence supports the notion that many inherited diseases are caused by a disruption of protein homeostasis instigated by disease-associated mutations [53], [54]. Unbalances in the coordination of the activity of ER folding, quality control, and degradation machineries can result in many human diseases related to defective protein maturation [55], [56], [57]. It is therefore of high therapeutic significance to decipher the signaling mechanisms as well as the protein machineries essential for maintaining normal protein homeostasis. Our demonstration that CLC-1 protein is subject to stringent conformation surveillance systems in the process of protein synthesis warrants future identification of the signals mediating the degradation of CLC-1 in various cellular compartments. Elucidation of these surveillance mechanisms in CLC-1′s biosynthetic pathway may shed light on novel therapeutic strategies for myotonia congenita.

## Materials and Methods

### CLC-1 Constructs

Human CLC-1 cDNA in the pcDNA3 vector (Invitrogen) was used to create the myotonia-associated A531V mutant by employing the QuickChange site-directed mutagenesis kit (Stratagene), followed by DNA sequence verification. To create myc-tagged CLC-1 constructs, the overlap PCR mutagenesis method was used to insert the myc-epitope sequence (EQKLISEEDL) between the residues G438 and D439, which are located in the extracellular linker between L and M helices. For N-terminal green fluorescent protein (GFP)-tagged constructs, CLC-1 cDNA was subcloned into the pEGFP-C1 (Clontech) vector.

### Cell Culture and DNA Transfection

Three types of cell lines were employed in this study: tsA201, human embryonic kidney (HEK) 293T, and COS-7 cells. tsA201 and HEK293T cells are derived from the same line of HEK293 cells (*293tsA1609neo*) stably transfected with the gene for SV40 large T-antigen [58], [59]. Cells were grown in Dulbecco’s modified Eagle’s medium (DMEM) supplemented with 2 mM glutamine, 10% heat-inactivated fetal bovine serum (Hyclone), 100 units/ml penicillin, and 50 µg/ml streptomycin, and were maintained at 37°C in a humidified incubator with 95% air and 5% CO_2_. Transient transfection was performed by using the Lipofectamine 2000 (LF2000) reagent (Invitrogen). Briefly, cells were plated onto 6-well plates (for biochemical experiments) or poly-D-lysine-coated coverslips in 24-well plates (for confocal imaging and electrophysiological recording) 24 hrs before transfection. Various expression constructs were incubated with LF2000 reagent for 20 min at room temperature, and DNA-lipofectamine diluted in Opti-MEM (Invitrogen) was added to culture wells containing the plated cells (1.05 µg total cDNA/12 mm coverslip). After 6-hr incubation at 37°C, the medium was changed and the culture cells were maintained in a 27°C or 37°C incubator for 24–48 hrs before being used for confocal imaging or for electrophysiological or biochemical experiments. Where indicated, drugs (MG132, cycloheximide, or NH_4_Cl) (Sigma) were applied to the culture medium.

### Electrophysiological Recordings

Conventional cell-attached, inside-out, or whole-cell patch-clamp techniques were employed to record CLC-1 Cl^−^ currents. Cells co-transfected with the cDNA for CLC-1 and pEGFP (molar ratio 3∶1) were identified with an inverted fluorescence microscope (Leica-DM IRB). Recording electrodes were pulled by a PP-830 puller (Narashige), and displayed a resistance of 1–2 MΩ when filled with the pipette solution. Both pipette and bath solutions contained (in mM): 130 NaCl, 5 MgCl_2_, 1 EGTA, 10 HEPES, pH 7.4. Data were acquired with an Axopatch 200B amplifier and digitized with the Digidata 1322A digitization board controlled by the pCLAMP 9.0 software (Molecular Devices). For whole-cell recordings, cell capacitances were measured using the built-in functions of the pCLAMP 9.0 software and were compensated electronically with the Axopatch 200B amplifier. The holding potential was set at 0 mV. Data were sampled at 2 kHz and filtered at 1 kHz. All recordings were performed at room temperature (20–22°C).

Electrophysiological experiments were conducted to obtain the voltage-dependence of the open probability (*P*_o_–V curve) and the instantaneous current-voltage (I-V) relationship. The voltage protocol has been described previously [60]. To estimate the *P*_o_ of the channel, the value of the initial tail-current, determined by fitting the tail-current to a double-exponential function, was normalized to the maximal initial tail-current obtained following the most positive test-voltage. Data points in the *P*_o_-V curve were fitted with a Boltzmann equation: *P*_o_ = *P_min_*+(1−*P_min_*)/{1+exp[zF(V−V_1/2_)/RT]}, where V_1/2_ is the half-activating voltage for the *P*_o_-V curve. To obtain the instantaneous I-V relationship, the relaxation process of the current elicited by the test-voltage was fitted to a double exponential function, and the initial current was determined by extrapolating the fitted exponential function to the beginning of the test-voltage. The measured instantaneous current was normalized to that measured at +80 mV, and the values from different patches were averaged to obtain the averaged I-V relationship.

### Immunoblotting

Two days after transfection, HEK293T cells were washed twice with ice-cold PBS and resuspended in a hypotonic buffer (10 mM Tris, pH 8.0) containing protease inhibitor cocktail (Roche Applied Science) and 2 mM EDTA. After adding Laemmli sample buffer to the lysates, samples were sonicated on ice (three times for five seconds each) and heated at 70°C for 5 min. Samples were then separated by 6% or 7.5% SDS-PAGE, electrophoretically transferred to nitrocellulose membranes, and detected using mouse anti-myc (clone 9E10) or mouse anti-β-actin (1∶5000; Sigma) antibodies. Blots were then exposed to horseradish peroxidase-conjugated anti-mouse IgG (1∶5000; Thermo Scientific), and revealed by an enhanced chemiluminescence detection system (Thermo Scientific). Data from multiple independent experiments were pooled together for quantification analyses by using the ImageJ software (National Institutes of Health). The apparent molecular weights of protein bands were calculated from the standard curves based on the mobility of molecular mass standards.

### Flow Cytometric Analyses

HEK293T cells transfected with cDNA for different GFP constructs (pEGFP, GFP-CLC-1 WT, or GFP-CLC-1 A531V) were harvested with trypsin/EDTA, washed twice with ice-cold PBS, and resuspended in PBS to the final concentration of 5×10^5^ cells/ml. Cells (10,000/sample) were then analyzed by the FACSCalibur flow cytometer system (BD Biosciences). The percentage of cells showing GFP fluorescence was quantitatively determined and was taken as an estimate of the cDNA transfection rate of each construct.

### Protein Ubiquitination Analyses

Transfected HEK293T cells were incubated at 37°C in the absence or presence of MG132 for 24 hrs. Cells were solubilized in ice-cold immunoprecipitation (IP) buffer [(in mM) 100 NaCl, 4 KCl, 2.5 EDTA, 20 NaHCO_3_, 20 Tris-HCl, pH 7.5, 1 phenylmethylsulfonyl fluoride, 1% Triton X-100] containing protease inhibitor cocktail (Roche Applied Science). Insolubilized materials were removed by centrifugation. Solubilized lysates were incubated for 16 hrs at 4°C with protein A-Sepharose beads (Pierce) previously coated with the anti-myc antibody. Beads were washed three times in IP buffer and twice with IP buffer with Triton X-100. The immune complexes were subject to immunoblotting with the anti-myc or anti-ubiqutin (FK2; Enzo Life Sciences) antibody.

### Biotinylation of Cell Surface Proteins

Transfected cells were washed extensively with PBS supplemented with 0.5 mM CaCl_2_, 2 mM MgCl_2_ (CM-PBS), followed by incubation in 1 mg/ml sulfo-NHS-LC-biotin (Thermo Scientific) in CM-PBS at 4°C for 30 min with gentle rocking. Biotinylation was terminated by removing the biotin reagents and rinsing three times each with CM-PBS and the Tris buffer saline (TBS)[(in mM) 20 Tris-HCl, 150 NaCl, pH 7.4]. Cells were solubilized in the lysis buffer [(in mM) 150 NaCl, 50 Tris-HCl, 1% Triton X-100, 5 EDTA, 1 phenylmethylsulfonyl fluoride, pH 7.6] supplemented with a protease inhibitor cocktail. Insolubilized materials were removed by centrifugation. Solubilized cell lysates were incubated overnight at 4°C with streptavidin-agarose beads (Thermo Scientific). Beads were washed three times in the lysis buffer and twice with TBS. The biotin-streptavidin complexes were eluted from the beads by heating at 70°C for 5 min in the Laemmli sample buffer, followed by SDS-PAGE and immunoblotting.

### Immunofluorescence

Transfected cells were rinsed in the phosphate buffer saline (PBS) [(in mM) 136 NaCl, 2.5 KCl, 1.5 KH_2_PO_4,_ 6.5 Na_2_HPO_4_, pH 7.4] and then fixed with 4% paraformaldehyde in PBS at 4°C for 20 min. Cells were washed three times with PBS and then blocked for 1 hr in PBS containing 0.1% (v/v) Triton X-100 and 5% normal goat serum. Cells were then incubated overnight at 4°C with the anti-myc antibody diluted in the blocking buffer. After three washes with PBS, the coverslips were incubated with goat-anti-rabbit/mouse antibodies conjugated to Alexa488 (Invitrogen) for 1 hr at room temperature. Nuclei were labeled with DAPI. Finally, the coverslips were rinsed once in blocking buffer, twice in PBS, and twice in 0.1 M carbonate buffer, pH 9.2, before they were mounted on glass slides in a mounting medium (4% n-propyl gallate, 90% glycerol, 0.1 M carbonate, pH 9.2). The fluorescence images of the fixed cultures were viewed and acquired with a Leica TCS SP5 laser-scanning confocal microscope.

### Statistical Analyses

All values were presented as mean ± SEM. The significance of the difference between two means was tested using the Student’s *t* test, whereas means from multiple groups were compared using the one-way ANOVA analysis. All statistical analyses were performed with the Origin 7.0 software (Microcal Software).

## Supporting Information

Figure S1 **Effects of the MG132 treatment on the A531V expression in COS-7 cells.** ***(A)*** Immunoblotting analyses of cell lysates from transfected COS-7 cells in the absence (*left*) or presence (*right*) of 20 µM MG132 for 24 hrs. ***(B)*** Quantification of CLC-1 protein expression levels. Protein densities were normalized to those of WT with no drug treatment.
(TIF)
