# Supplementary figures and images for: Myotonia Congenita Mutation Enhances the Degradation of Human CLC-1 Chloride Channels
Source: PLoS One. 2013 Feb 12;8(2):e55930. doi: 10.1371/journal.pone.0055930 (PMC3570542; doi:10.1371/journal.pone.0055930)

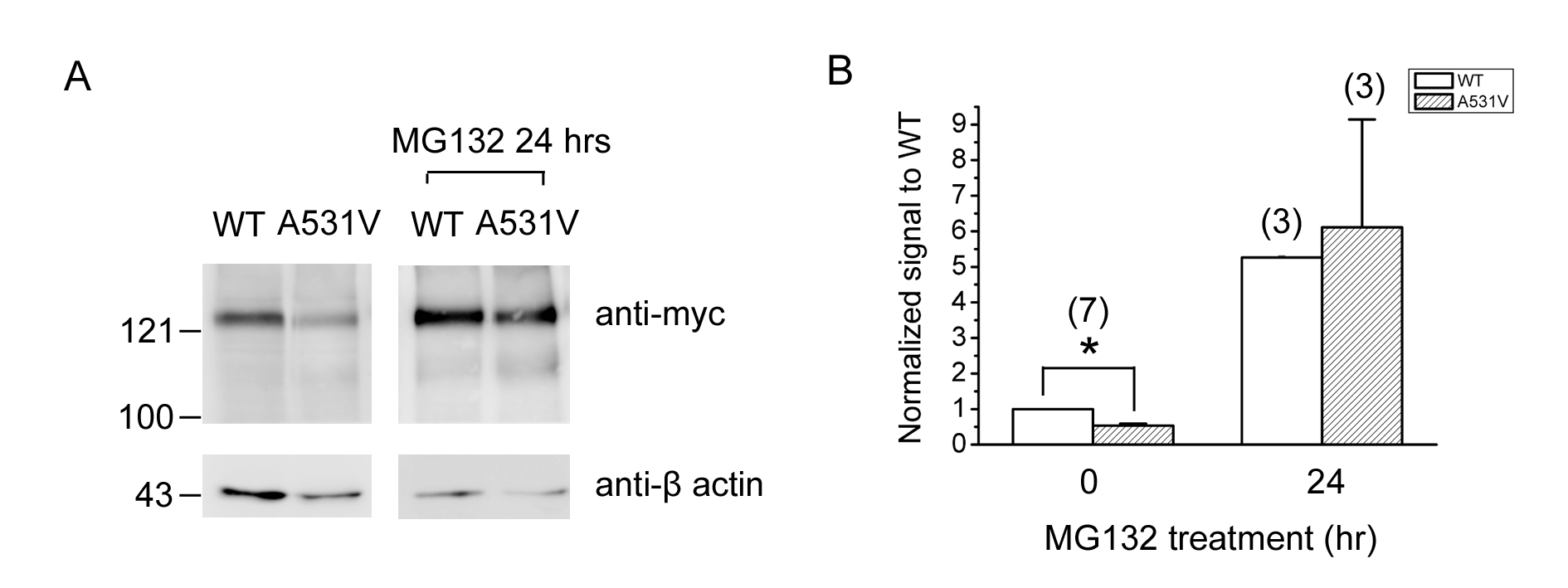

Supplement: Figure S1 — Effects of the MG132 treatment on the A531V expression in COS-7 cells. (A) Immunoblotting analyses of cell lysates from transfected COS-7 cells in the absence (left) or presence (right) of 20 µM MG132 for 24 hrs. (B) Quantification of CLC-1 protein expression levels. Protein densities were normalized to those of WT with no drug treatment. (TIF) [file pone.0055930.s001.tif]
